# Vitamin D monitoring and management in the inpatient services – reaudit

**DOI:** 10.1192/bjo.2021.266

**Published:** 2021-06-18

**Authors:** Vasudevan Krishnan, Ciara Doyle, Maciej Rusilowicz

**Affiliations:** 1 Midlands Partnership NHS Trust; 2 University Hospitals of Derby and Burton NHS Foundation Trust

## Abstract

**Aims:**

To survey the prevalence of monitoring of vitamin D on an inpatient ward.

To audit the treatment if there is identified vitamin D deficiency or insufficiency

To compare differences between findings in audits

**Method:**

All inpatients admitted to Milford centre between August 2019 and August 2020 were selected as part of the sample size.

Data were collected by FY1 and FY2

Patients’ laboratory results were accessed to determine vitamin D levels.

E-notes were used to conclude who were vitamin D sufficient or deficient for treatment

The standard for the audit were as per:

Management of vitamin D deficiency or insufficiency in adults – CKS (2018)

The above was based on National Osteoporosis Society (NOS) guideline *Vitamin D and bone health: a practical clinical guideline for patient management* [National Osteoporosis Society, 2013] and Scientific Advisory Committee on Nutrition (SACN) guideline

**Result:**

2017

48/188 patients had vitamin D levels measured

36/48 patients had sufficient vitamin D levels

12/48 patients were either deficient or insufficient

12/12 patients were treated where found deficient or insufficient

2020

90/115 patients had vitamin D levels measured

47/90 patients had sufficient vitamin D Levels

43/90 patients had either insufficient or deficient vitamin D levels

22/43 patients had treatment documented in noted where found deficient or insufficient

**Conclusion:**

Difficult to make comparisons with previous audit due to difference in number of patients tested

Vitamin D is routinely tested on Milford ward on admission hence the large number compared to the last audit

52% had noted to have sufficient levels of vitamin D

Concerning were results that only 51% of those deemed to have insufficient or deficient were treated based on notes

Potential reasons could be:

Prescribed in medication card and not documented in notes.

Vitamin D results checked in another ward, no supplementation given, and then transferred to Milford house.

Patients refused treatment but not documented adequately.

Patient discharged before results were received due to quick around

Results were deemed insufficient in terms of the range but very close to normal hence decision made not to start supplementation

Results to be disseminated with medical and nursing colleagues

Re-audit in September 2021

